# Sex differences in the nonlinear association of triglyceride glucose index with all-cause and cardiovascular mortality in the general population

**DOI:** 10.1186/s13098-023-01117-7

**Published:** 2023-06-23

**Authors:** Yu Yu, Jing Wang, Ligang Ding, Hao Huang, Sijing Cheng, Yu Deng, Min Gu, Chi Cai, Xiaohui Ning, Xuhua Chen, Hongxia Niu, Wei Hua

**Affiliations:** Cardiac Arrhythmia Center, Department of Cardiology, National Center for Cardiovascular Diseases, State Key Laboratory of Cardiovascular Disease, Fuwai Hospital, Chinese Academy of Medical Sciences and Peking Union Medical College, Beijing, China

**Keywords:** Triglyceride glucose index, Insulin resistance, Mortality, Sex difference, NHANES

## Abstract

**Background:**

The evidence on the association between the triglyceride glucose (TyG) index and the risk of death in the general population remains controversial. This study aims to investigate the relationship between the TyG index and all-cause and cardiovascular mortality in the general population, with a focus on sex differences.

**Methods:**

This prospective cohort study analyzed data from the National Health and Nutrition Examination Survey (1999–2002), comprising 7,851 US adults. The study employed multivariate Cox proportional hazards regression and two-segment Cox hazard regression models to evaluate the sex-specific differences in the relationship between the TyG index and all-cause and cardiovascular mortality.

**Results:**

After 11,623 person-years of follow-up, there were 539 deaths, with 10.56% due to all-cause mortality and 2.87% due to cardiovascular mortality. After adjusting for multiple variables, our study found a U-shaped association of the TyG index with all-cause and cardiovascular mortality, with inflection points at 9.36 and 9.52. A significant sex difference was observed in the association between the TyG index and mortality. Below the inflection point, the relationship between the TyG index and mortality was consistent in males and females. However, above the inflection point, only males exhibited a positive association between the TyG index and all-cause mortality (adjusted hazard risk [HR], 1.62, 95% confidence interval [CI], 1.24–2.12) and cardiovascular mortality (adjusted HR, 2.28, 95% CI, 1.32–3.92).

**Conclusions:**

Our study showed a U-shaped association between the TyG index and all-cause and cardiovascular mortality in the general population. Furthermore, sex differences were observed in the association between the TyG index and mortality once it exceeded a certain threshold.

## Introduction

Insulin resistance (IR) is a key feature of type 2 diabetes (T2DM) and significantly contributes to the development of metabolic and cardiovascular diseases (CVD) [1]. The triglyceride glucose (TyG) index is a reliable surrogate biomarker of IR that was proposed in 2008 and has gained recognition as a simple and cost-effective indicator [2, 3]. Despite several studies investigating the prognostic value of the TyG index for survival benefits in the general population, existing research has yielded inconsistent conclusions regarding its association with all-cause and cardiovascular mortality [4, 5]. Some studies have reported an inverse association between the TyG index and all-cause mortality, while others have found a positive or no association [6–9]. Similarly, studies examining the relationship between the TyG index and cardiovascular mortality have yielded conflicting results [6–8]. These controversial findings have limited the clinical application of the TyG index. Therefore, a better understanding of the relationship between the TyG index and all-cause and cardiovascular mortality is crucial to promoting the use of the TyG index in clinical practice and improving survival rates in the general population.

The risk of death is known to vary between sexes throughout life and widens with age, potentially due to differences in body composition, lifestyle, and genetics [10, 11]. Therefore, when making clinical decisions related to survival benefits, the effect of sex should be taken into consideration. Additionally, gender also affects the degree of IR, with impaired fasting glucose being more prevalent in women [12, 13]. As such, the relationship between the TyG index, which indicates IR, and mortality may differ between sexes to varying degrees. However, the specific effect of sex on this relationship is poorly understood due to a lack of direct evidence.

Therefore, we aimed to investigate the association between the TyG index and all-cause and cardiovascular mortality, focusing on the potential modifying effect of sex. Our study may address the research gap and mitigate sex-related bias in IR treatment for improved survival outcomes.

## Methods

### Study design and population

**Fig. 1:**
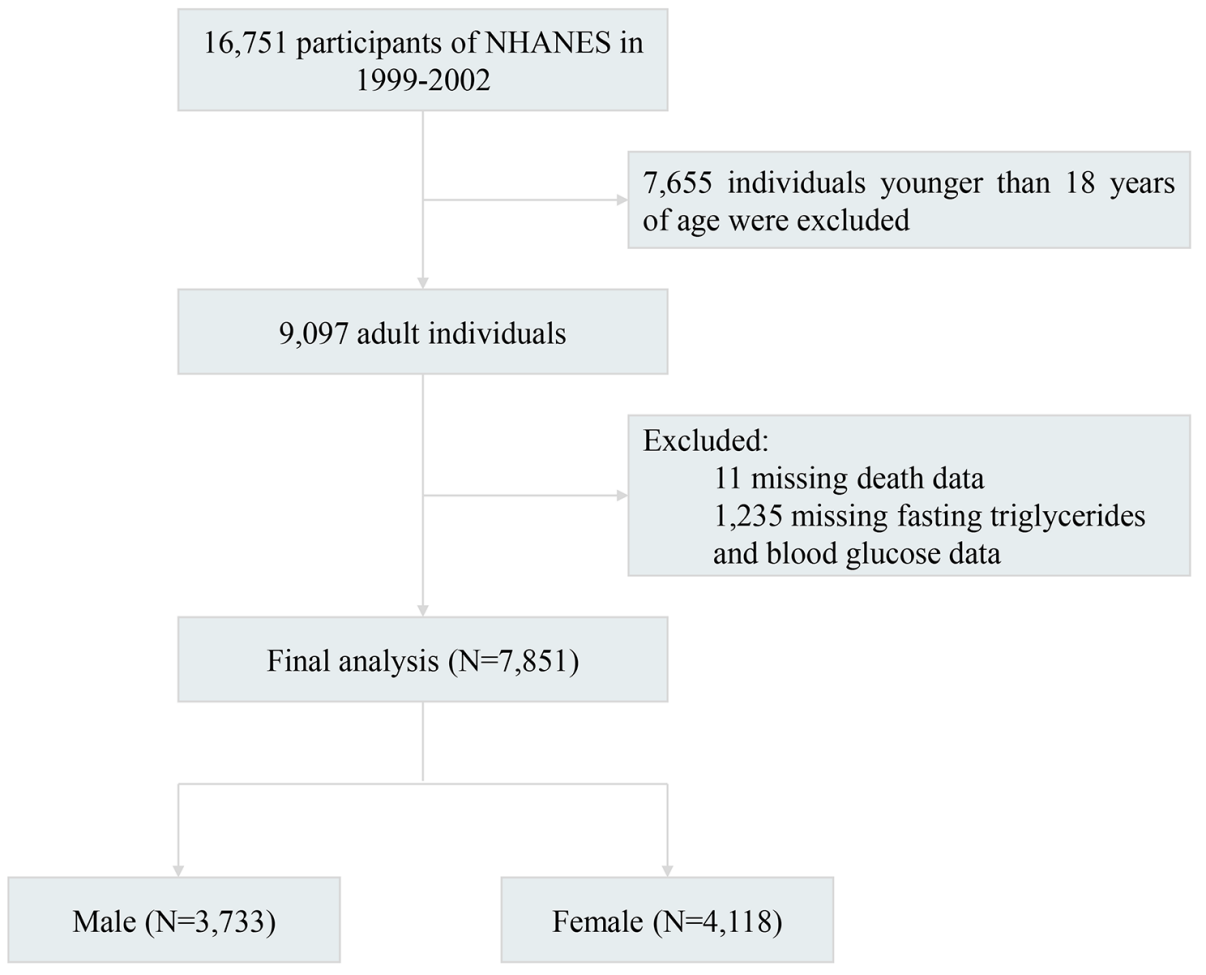
Flow chart of study participants

The National Health and Nutrition Examination Survey (NHANES) is a research project conducted by the Centers for Disease Control and Prevention (CDC) and the National Centers for Health Statistics (NCHS) to assess the health and nutritional status of adults and children in the United States. The NCHS Research Ethics Review Committee approved the NHANES study protocol, and all participants signed written informed consent. This study followed the Strengthening the Reporting of Observational Studies in Epidemiology (STROBE) reporting guideline for cohort studies. Of the 16,751 participants initially included in two survey cycles of NHANES (1999–2000 and 2001–2002), we excluded 7,655 individuals under the age of 18 years, leaving 9,097 individuals aged ≥ 18 years. After excluding 11 missing deaths and 1,235 individuals with missing triglyceride and glucose data, a total of 7,851 patients were included in the study. The flowchart of the study population is visualized in Fig. 1.

### Covariates

The covariates in this study consisted of demographic characteristics, physical examination, disease history, and laboratory tests. Standard questionnaires assessed sociodemographic characteristics and disease history. Demographic characteristics included age, sex, education, physical activity, smoking, and drinking status. Information on diabetes, hypertension, coronary atherosclerotic heart disease (CAD), cancer, and medication was self-reported. Study participants’ height, weight, waist circumference, and blood pressure were tested at the Mobile Examination Center. Fasting blood glucose (FBG) concentrations were measured using a complete blood count identification procedure. Glycosylated hemoglobin (HbA1c) was obtained by analyzing whole blood samples using a Primus automated HPLC system (Primus I, model CLC330). The values in total cholesterol (TC), triglycerides (TG), low-density lipoprotein cholesterol (LDL-C), and high-density lipoprotein cholesterol (HDL-C) were obtained by analyzing fasting venous blood samples using Hitachi 704 analyzer. The estimated glomerular filtration rate (eGFR) was calculated by the simplified MDRD formula [14]. TyG index was calculated with the following formula: TyG index = Ln [fasting TG (mg/dL) × FBG (mg/dL)/2] [15]. More details of covariates measurements are described on the NHANES website (https://www.cdc.gov/nchs/nhanes/index.htm).

### Ascertainment of mortality

The study outcome was all-cause mortality. All-cause mortality included heart disease (I00-I09, I11, I13, I20-I51), cerebrovascular disease (I60-I69), cancer (C00-C97), and respiratory disease (J10-J18, J40-J47) causes of death. The NHANES participants in the study were followed up for mortality through December 31, 2006. Death data were extracted from public-use linked mortality files in the NHANSE database.

### Statistical analysis

All statistical analyses were conducted in accordance with CDC guidelines (https://wwwn.cdc.gov/nchs/nhanes/tutorials/default.aspx). Since NHANES employs a complex multistage stratified probability survey design, the statistical analysis in this study incorporated sample weights, clustering, and stratification. Baseline characteristics of the study population were stratified by sex and TyG index tertiles, with continuous variables presented as survey-weighted mean and categorical variables presented as survey-weighted percentage (%). Multivariable Cox proportional hazards regression models were used to explore the sex-specific association of the TyG index with all-cause and cardiovascular mortality, with adjustment variables selected based on clinical relevance and candidate variables with a P value < 0.05 in univariate analysis [2]. Generalized additive models and penalized spline methods were used to examine the associations between the TyG index and mortality risk. If the P for log-likelihood ratio was less than 0.05, then the inflection point was identified using a recursive algorithm; and a two-segment Cox proportional hazards model was used to calculate hazard risk (HR) values and 95% CIs for the relationship on both sides of the inflection point.

All analyses were performed using the statistical packages R version 4.0.2 and SPSS (IBM) version 23, and a two-tailed P < 0.05 was considered statistically significant.

## Results

### Baseline characteristics of study participants

A total of 7851 adults were included in the final analysis (mean age, 46.16 ± 20.16 years; 3733 (47.5%) were men). The weighted mean blood glucose was 5.39 ± 1.96 mmol/L, the mean TG was 1.59 ± 1.51 mmol/L, and the mean TyG index was 8.61 ± 0.69. The baseline characteristics of the study population stratified by sex were presented in Table 1. Compared to female participants, male participants were more likely to be younger, more physical activity, higher rates of smoking and alcohol consumption, and higher proportions of diabetes, CAD, and higher levels of TyG index, glucose, HbA1c, TG, uric acid, and eGFR at baseline (*P* < 0.01). In addition, the baseline characteristics stratified by tertiles of the TyG index were displayed in Table 2, patients in the T2 group were younger, more physically active, had lower rates of smoking and alcohol consumption, lower obesity levels, lower blood pressure, and a lower incidence of diabetes, hypertension, CAD, and cancer compared to those in the T1 and T2 TyG groups.

**Table 1:** Baseline characteristics of the study population stratified by sex

Characteristics	Overall		Male		Female		P-value
Age, years	7851	(45.1)	3733	(44.3)	4118	(45.8)	0.001
Education, %							0.995
< High school	2680	(21.8)	1324	(21.8)	1356	(21.8)	
High school	1842	(25.4)	853	(25.3)	989	(25.4)	
> High school	3312	(52.8)	1549	(52.9)	1763	(52.7)	
Physical activity, %							< 0.001
Inactive	3981	(48.2)	1727	(44)	2254	(52)	
Active	3316	(51.8)	1669	(56)	1647	(48)	
Smoking, %							< 0.001
Never	3665	(50.5)	1366	(43)	2299	(57.4)	
Ever or current	3387	(49.5)	1973	(57)	1414	(42.6)	
Drinking, %	936	(16.4)	755	(24.6)	181	(7.5)	< 0.001
BMI, kg/m2	7564	(27.9)	3591	(27.8)	3973	(28.1)	0.064
WC, cm	7579	(95.6)	3609	(98.8)	3970	(92.7)	< 0.001
SBP, mmHg	6245	(118.6)	3031	(121.5)	3214	(115.8)	< 0.001
DBP, mmHg	6245	(71.3)	3031	(72.8)	3214	(69.9)	< 0.001
Diabetes, %	804	(7.7)	421	(8.4)	383	(6.9)	0.009
Hypertension, %	2192	(25.7)	1013	(24)	1179	(27.2)	0.026
CAD, %	288	(3.5)	186	(4.4)	102	(2.6)	0.009
Cancer, %	587	(7.9)	295	(6.9)	292	(8.9)	0.002
Antihypertensive drugs, %	1384	(14.9)	666	(13.8)	718	(16)	0.039
Statins, %	505	(6.5)	274	(7)	231	(6)	0.244
Glucose-lowering drugs, %	425	(3.8)	204	(3.8)	221	(3.7)	0.827
TyG index	7851	(8.6)	3733	(8.7)	4118	(8.5)	< 0.001
Glucose, mmol/L	7851	(5.3)	3733	(5.4)	4118	(5.2)	< 0.001
HbA1c, %	7846	(5.5)	3729	(5.5)	4117	(5.4)	0.002
TC, mmol/L	7836	(5.2)	3724	(5.2)	4112	(5.3)	0.050
TG, mmol/L	7851	(1.6)	3733	(1.8)	4118	(1.4)	< 0.001
LDL-C, mmol/L	7836	(3.1)	3724	(3.2)	4112	(3.1)	0.091
HDL-C, mmol/L	7836	(1.3)	3724	(1.2)	4112	(1.5)	< 0.001
UA, umol/L	7850	(319.0)	3732	(362.7)	4118	(278.4)	< 0.001
eGFR, ml/min/1.73m2	7850	(105.0)	3732	(128.5)	4118	(83.1)	< 0.001

Data in the table are presented as survey-weighted mean or survey-weighted percentage (%)

BMI: body mass index; WC: waist circumference; SBP: systolic blood pressure; DBP: diastolic blood pressure; CAD: coronary atherosclerotic heart disease; TyG: triglyceride glucose; HbA1c: glycated haemoglobin; TC: total cholesterol; TG: triglycerides; LDL-C: low-density lipoprotein cholesterol; HDL-C: high-density lipoprotein cholesterol; UA: uric acid; eGFR: estimated glomerular filtration rate

**Table 2:** Baseline characteristics of the study population stratified by TyG index tertiles

Characteristics	T 2 (8.28–8.83)		T 1 (6.56–8.28)		T 3 (8.83–13.3)		P-value
Age, years	2614	(39.2)	2619	(46.1)	2618	(50.2)	< 0.001
Education, %							< 0.001
< High school	797	(18.6)	888	(21.5)	995	(25.6)	
High school	619	(23.6)	633	(26.6)	590	(26)	
> High school	1196	(57.9)	1088	(51.8)	1028	(48.5)	
Physical activity, %							< 0.001
Inactive	1172	(41.2)	1345	(49.3)	1464	(54.4)	
Active	1243	(58.8)	1080	(50.7)	993	(45.6)	
Smoking, %							< 0.001
Never	1202	(54.9)	1262	(50.1)	1201	(46.4)	
Ever or current	932	(45.1)	1145	(49.9)	1310	(53.6)	
Drinking, %	241	(14.7)	304	(15.5)	391	(19.2)	< 0.001
BMI, kg/m2	2546	(25.4)	2510	(28.3)	2508	(30.2)	< 0.001
WC, cm	2543	(87.3)	2513	(96.7)	2523	(103.4)	< 0.001
SBP, mmHg	2262	(115.3)	2061	(118.9)	1922	(122.5)	< 0.001
DBP, mmHg	2262	(70)	2061	(71.6)	1922	(72.7)	< 0.001
Diabetes, %	62	(1.5)	157	(4.2)	585	(17.8)	< 0.001
Hypertension, %	459	(14.5)	752	(26.5)	981	(36.7)	< 0.001
CAD, %	39	(1.3)	95	(3.2)	154	(5.9)	< 0.001
Cancer, %	143	(6.2)	209	(8.7)	235	(8.9)	0.01
Antihypertensive drugs, %	231	(6.3)	481	(15.8)	672	(23)	< 0.001
Statins, %	161	(6.1)	62	(2.4)	282	(11.3)	< 0.001
Glucose-lowering drugs, %	82	(2)	39	(0.7)	304	(8.8)	< 0.001
TyG index	2619	(8.6)	2614	(7.9)	2618	(9.4)	< 0.001
Glucose, mmol/L	2619	(5)	2614	(4.8)	2618	(6.1)	< 0.001
HbA1c, %	2618	(5.3)	2613	(5.2)	2615	(5.9)	< 0.001
TC, mmol/L	2611	(5.3)	2612	(4.7)	2613	(5.7)	< 0.001
TG, mmol/L	2619	(1.3)	2614	(0.7)	2618	(2.9)	< 0.001
LDL-C, mmol/L	2611	(3.3)	2612	(2.9)	2613	(3.2)	< 0.001
HDL-C, mmol/L	2611	(1.3)	2612	(1.5)	2613	(1.1)	< 0.001
UA, umol/L	2619	(321.4)	2614	(288.6)	2617	(348.6)	< 0.001
eGFR, ml/min/1.73m2	2619	(102.2)	2614	(104.2)	2617	(108.7)	0.07

Data in the table are presented as survey-weighted mean or survey-weighted percentage (%)

BMI: body mass index; WC: waist circumference; SBP: systolic blood pressure; DBP: diastolic blood pressure; CAD: coronary atherosclerotic heart disease; TyG: triglyceride glucose; HbA1c: glycated haemoglobin; TC: total cholesterol; TG: triglycerides; LDL-C: low-density lipoprotein cholesterol; HDL-C: high-density lipoprotein cholesterol; UA: uric acid; eGFR: estimated glomerular filtration rate

### Sex-specific association between the TyG index and all-cause mortality

**Fig. 2:**
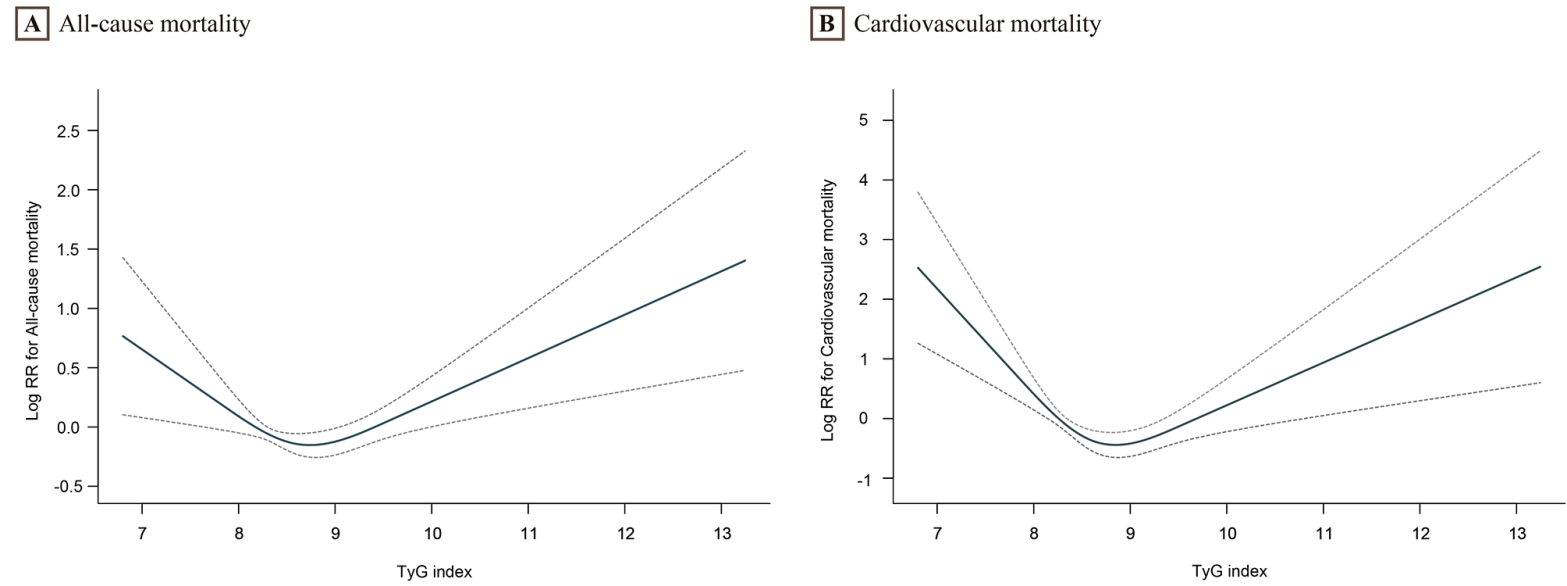
Associations between TyG index and all-cause **(A)** and cardiovascular mortality **(B)** in all participants The solid line and dashed line represent the estimated values and their corresponding 95% confidence interval. Adjusted for survey cycle, age, sex, education, smoking, BMI, LDL-C, HDL-C, eGFR, diabetes, hypertension, CAD, cancer, statins, and glucose-lowering drugs

**Fig. 3:**
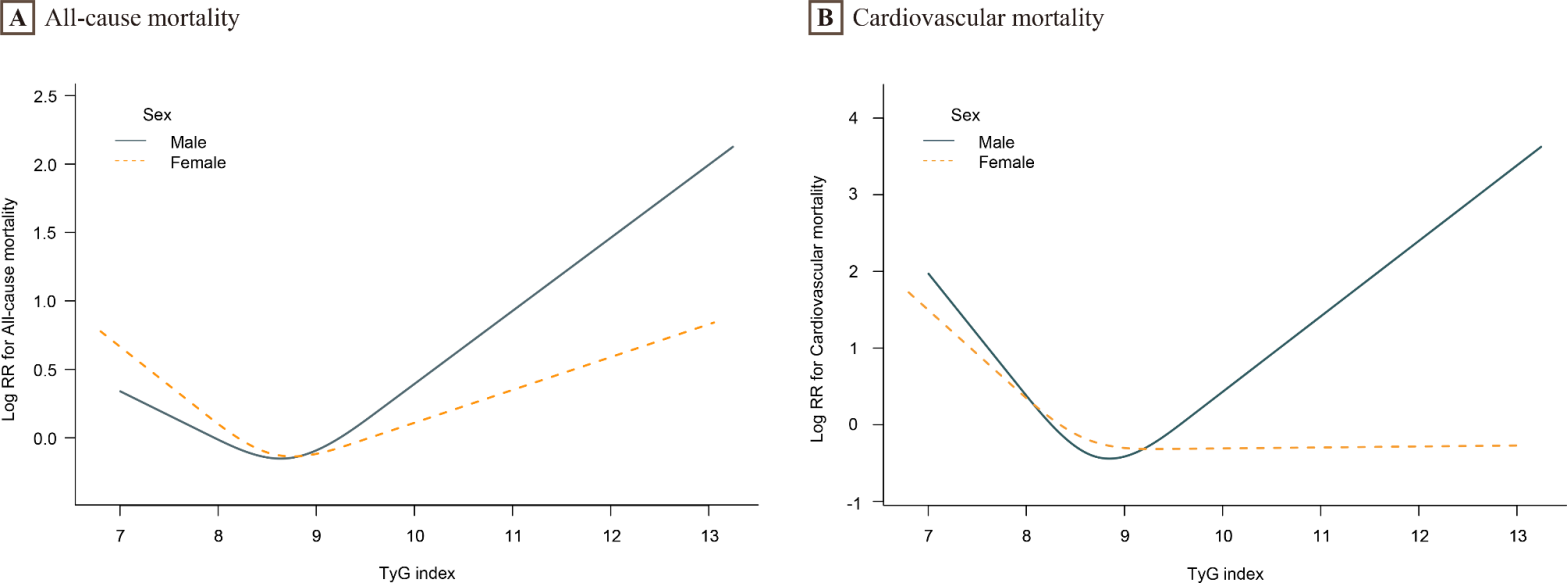
Association of TyG index with all-cause and cardiovascular mortality was evaluated in males **(A)** and females **(B)** The solid line represents males, and the dashed line represents females. Adjusted for survey cycle, age, education, smoking, BMI, LDL-C, HDL-C, eGFR, diabetes, hypertension, CAD, cancer, statins, and glucose-lowering drugs

During the 11,623 person-years of follow-up, 539 deaths occurred. The TyG index was categorized into three groups based on tertiles (Table 3). After multivariate adjustment, the T3 group had a higher risk of all-cause mortality than the T2 (8.28–8.83) group (reference) in all participants (HR, 1.38; 95%CI, 1.08–1.77). Similarly, in males, the T1 and T3 groups had higher HRs (95% CI) for all-cause mortality compared to the T2 (8.35–8.91) group as a reference: 1.52 (1.03, 2.24) and 2.14 (1.51, 3.01), respectively. In female participants, the T1 group had a higher risk of all-cause mortality than the T2 group (HR, 1.56; 95%CI, 1.06–2.44). Figure 2 A revealed a U-shaped relationship between the TyG index and all-cause mortality, with an inflection point of 8.5. The HR (95%CI) for the left side of the inflection point (TyG < 8.5) was 0.55 (0.34, 0.90), and for the right side (TyG ≥ 8.5) was 1.39 (1.14, 1.70) (Table 4). Figure 3 A shows that the U-shaped relationship between the TyG index and all-cause mortality exists in males and females, with an inflection point of 8.5. We observed no significant relationship between the TyG index and all-cause mortality in males and females when the TyG index was lower than 8.5. However, when TyG ≥ 8.5, a positive association between the TyG index and all-cause mortality was observed in males (HR, 1.62; 95%CI, 1.24–2.12), but not in females.

**Table 3:** Association of TyG index with all-cause and cardiovascular mortality in the total population and stratified by sex

TyG index	Age-standardized mortality rate, %	HR (95% CI), P-value					
		Model 1		Model 2		Model 3	
***All-cause mortality***							
**All participants**							
Tertiles							
T 1 (6.56–8.28)	10.00%	1.23 (0.97, 1.56)	0.081	1.24 (0.93, 1.65)	0.136	1.24 (0.93, 1.66)	0.142
T 2 (8.28–8.83)	7.92%	1 [Reference]		1 [Reference]		1 [Reference]	
T 3 (8.83–13.3)	13.01%	1.33 (1.10, 1.62)	0.004	1.47 (1.16, 1.87)	0.001	1.38 (1.08, 1.77)	0.01
**Males**							
Tertiles							
T 1 (6.96–8.35)	11.92%	1.51 (1.12, 2.03)	0.006	1.48 (1.02, 2.16)	0.038	1.52 (1.03, 2.24)	0.034
T 2 (8.35–8.91)	11.06%	1 [Reference]		1 [Reference]		1 [Reference]	
T 3 (8.91–13.3)	15.47%	1.64 (1.26, 2.13)	< 0.001	2.05 (1.47, 2.85)	< 0.001	2.14 (1.51, 3.01)	< 0.001
**Females**							
Tertiles							
T 1 (6.56–8.22)	8.10%	1.32 (0.90, 1.94)	0.151	1.46 (0.94, 2.27)	0.093	1.56 (1.06, 2.44)	0.041
T 2 (8.22–8.77)	5.64%	1 [Reference]		1 [Reference]		1 [Reference]	
T 3 (8.77–13.05)	10.42%	1.21 (0.89, 1.64)	0.229	1.15 (0.81, 1.65)	0.431	1.07 (0.73, 1.54)	0.738
***Cardiovascular mortality***							
**All participants**							
Tertiles							
T 1 (6.56–8.55)	2.69%	1.35 (0.92, 1.97)	0.126	1.37 (0.85, 2.22)	0.2	1.46 (0.89, 2.39)	0.135
T 2 (8.55–9.35)	2.63%	1 [Reference]		1 [Reference]		1 [Reference]	
T 3 (9.35–13.3)	3.89%	1.44 (0.93, 2.23)	0.105	1.47 (0.85, 2.55)	0.168	1.35 (0.74, 2.47)	0.327
**Males**							
Tertiles							
T 1 (6.95–8.55)	3.88%	1.31 (0.82, 2.12)	0.262	1.35 (0.72, 2.55)	0.351	1.56 (0.81, 3.02)	0.185
T 2 (8.55–9.35)	3.32%	1 [Reference]		1 [Reference]		1 [Reference]	
T 3 (9.35–13.3)	4.10%	1.35 (0.78, 2.36)	0.282	1.57 (0.78, 3.15)	0.206	1.78 (0.83, 3.83)	0.14
**Females**							
Tertiles							
T 1 (6.56–8.55)	1.96%	1.35 (0.72, 2.54)	0.353	1.24 (0.53, 2.48)	0.739	1.17 (0.48, 2.40)	0.866
T 2 (8.55–9.35)	1.75%	1 [Reference]		1 [Reference]		1 [Reference]	
T 3 (9.35–13.3)	3.62%	1.56 (0.76, 3.21)	0.23	1.16 (0.46, 2.92)	0.751	0.78 (0.26, 2.28)	0.644

Model 1: Adjusted for survey cycle, age

Model 2: Adjusted for survey cycle, age, sex (only for all participants), education, smoking, BMI, LDL-C, HDL-C, and eGFR.

Model 3: Adjusted for survey cycle, age, sex (only for all participants), education, smoking, BMI, LDL-C, HDL-C, eGFR, diabetes, hypertension, CAD, cancer, statins, and glucose-lowering drugs

**Table 4:** Threshold effect analysis of TyG index on all-cause and cardiovascular mortality in the total population and stratified by sex

	Adjusted HR (95% CI), P-value					
	All participants		Males		Females	
**All-cause mortality**						
Fitting by the standard linear model	1.16 (0.98, 1.37)	0.092	1.32 (1.05, 1.67)	0.019	1.04 (0.81, 1.35)	0.744
Fitting by the two-piecewise linear model						
Inflection point	8.5		8.5		8.5	
TyG < 8.5	0.55 (0.34, 0.90)	0.016	0.59 (0.31, 1.13)	0.112	0.55 (0.26, 1.16)	0.116
TyG ≥ 8.5	1.39 (1.14, 1.70)	0.001	1.62 (1.24, 2.12)	0.001	1.24 (0.91, 1.70)	0.173
P for Log-likelihood ratio	0.002		0.011		0.044	
**Cardiovascular mortality**						
Fitting by the standard linear model	1.00 (0.67, 1.48)	0.989	1.25 (0.77, 2.02)	0.374	0.69 (0.34, 1.41)	0.311
Fitting by the two-piecewise linear model						
Inflection point	8.7		8.7		8.7	
TyG < 8.7	0.30 (0.14, 0.65)	0.002	0.28 (0.10, 0.76)	0.013	0.47 (0.12, 1.77)	0.263
TyG ≥ 8.7	1.82 (1.14, 2.92)	0.012	2.28 (1.32, 3.92)	0.003	0.93 (0.32, 2.75)	0.901
P for Log-likelihood ratio	< 0.001		0.002		0.045	

Adjusted for survey cycle, age, sex (only for all participants), education, smoking, BMI, LDL-C, HDL-C, eGFR, diabetes, hypertension, CAD, cancer, statins, and glucose-lowering drugs

### Sex-specific association between the TyG index and cardiovascular mortality

During 11,623 person-years of follow-up, 139 cardiovascular deaths occurred. Participants, including males and females, were divided into three tertiles (Table 3). In the fully adjusted model, there were no statistically significant differences in the risk of cardiovascular mortality between the T1 and T3 groups compared to the T2 group in the total population and gender-stratified subgroups (P > 0.05). Figure 2B illustrates a U-shaped relationship between the TyG index and cardiovascular mortality, with an inflection point of 8.7. Table 4 shows that the HR (95%CI) of cardiovascular mortality was 0.30 (0.14, 0.65) for TyG < 8.7 and 1.82 (1.14, 2.92) for TyG ≥ 8.7. In males, the relationship between the TyG index and all-cause mortality was U-shaped, and in females, it was L-shaped, both with inflection points of 8.7 (Fig. 3B). For males, each unit increase in TyG below 8.7 was associated with a 72% reduction in the risk of cardiovascular mortality (HR, 0.28; 95% CI, 0.10–0.76). However, when TyG was greater than or equal to 8.7, the TyG index was positively associated with cardiovascular mortality (HR, 2.28; 95% CI, 1.32–3.92). In contrast, there was no association between the TyG index and cardiovascular mortality in females, regardless of whether the TyG exceeded 8.7.

## Discussion

The present study utilized data from a nationally representative US prospective cohort to investigate the sex-specific relationship between the TyG index and all-cause and cardiovascular mortality. Our main findings indicate that the U-shaped associations between the TyG index and all-cause and cardiovascular mortality were observed in the general population, with a threshold of 8.5 for all-cause mortality and 8.7 for cardiovascular mortality. We further observed that sex modifies the association of the TyG index with mortality. Specifically, after exceeding a certain threshold, a positive association between the TyG index and all-cause and cardiovascular mortality was found in males but not females.

Several studies have investigated the relationship between the TyG index and mortality in the general population prior to our study. Kim KS et al. [16] observed an increased risk of all-cause (HR, 1.21; 95% CI, 1.14–1.28) and cardiovascular mortality (HR, 1.45; 95% CI, 1.26–1.66) in 255,508 healthy individuals with a mean follow-up of 5.7 years. However, Kim J et al. [9] found no significant association between the TyG index and cardiovascular or all-cause mortality in males (HR, 1.167; 95%CI, 0.698–1.954) and females. Conversely, Liu et al. [8] found a negative association between the TyG index and death in the general population, with thresholds of 9.36 and 9.52 for all-cause and cardiovascular mortality. The results were inconclusive due to differences in sample size, follow-up time, and target population. Our study determined a U-shaped relationship between the TyG index and all-cause and cardiovascular mortality in the general population, with thresholds of 8.5 and 8.7. Sun et al. [17] found similar results in 9,254 participants with age ≥ 45 years, demonstrating a U-shaped association of the TyG index with all-cause and cardiovascular mortality, with an inflection point of 9.18. The findings suggest that maintaining TyG levels in the appropriate range may provide a survival benefit, which could be a potential therapeutic strategy for reducing the risk of death in the general population.

Sex differences in the relationship between the TyG index and mortality were observed in this study, particularly above a certain threshold. Previous studies investigating the effect of sex on the association between the TyG index and unfavorable clinical events have yielded inconsistent results. For example, Lu et al. [18] found that the TyG index was a risk factor for subclinical atherosclerosis in males but not females, while Yang et al. [19] did not find a sex-specific effect of the TyG index on adverse cardiovascular events. Hong et al. [4] observed that a higher TyG index was associated with a greater risk of stroke and myocardial infarction in both men and women. However, there is a lack of direct research evidence on sex differences in the TyG index and all-cause and cardiovascular mortality. Consequently, our finding of a sex-specific threshold for TyG index and mortality has significant clinical implications for developing sex-specific management strategies to reduce mortality risk.

Given the effect of gender on IR and mortality risk, clarifying the effect of gender on the association between the TyG index and mortality can be beneficial in reducing mortality risk [10, 11]. Our study found a threshold for the TyG index and mortality risk, which is related to the cause of death and not gender. However, above this threshold, there was a significant sex inconsistency in the association between the TyG index and all-cause and cardiovascular mortality, with an unfavorable association observed in men but not women. This determination of the threshold of the TyG index level holds practical clinical significance. Relevant clinical treatment strategies for individuals with a TyG index above the threshold should consider sex-related differences. Nonetheless, below this threshold, gender may have little impact on the association between the TyG index and death.

The TyG index can be a valuable clinical tool for predicting adverse outcomes, offering an alternative to relying solely on glucose and triglyceride levels. This is supported by several arguments. First, TyG Index as a surrogate measure of IR. IR plays a crucial role in the pathophysiology of various metabolic disorders, including cardiovascular diseases [1]. Several studies have shown a strong correlation between the TyG index and gold standard measures of IR, such as the euglycemic-hyperinsulinemic clamp technique [20]. By incorporating both glucose and triglyceride levels into a single index, the TyG index offers a simple and cost-effective alternative for assessing IR in clinical practice. Elevated TyG index levels have consistently been associated with an increased risk of cardiovascular events and mortality. The TyG index reflects an imbalance in lipid and glucose metabolism, capturing the combined effect of hyperglycemia and dyslipidemia [21]. By considering the TyG index, clinicians can obtain additional prognostic information beyond what glucose and triglyceride levels alone can provide. Our study specifically focused on investigating the U-shaped association between the TyG index and all-cause and cardiovascular mortality in the general population, with a particular emphasis on sex differences. We found that both low and high TyG index values were associated with increased mortality risk compared to the mid-range values. This U-shaped pattern suggests that both hypoglycemia and IR, represented by low and high TyG index values, respectively, may contribute to adverse health outcomes. By considering the TyG index, clinicians can identify individuals at both ends of the spectrum who may benefit from targeted interventions aimed at improving metabolic balance.

Several possible mechanisms that could explain the observed U-shaped association between TyG and all-cause and cardiovascular mortality and their sex differences in the general population. These mechanisms are supported by multiple studies and include: (1) IR and Lipotoxicity: Elevated TyG levels have been associated with IR, which can lead to dyslipidemia, systemic inflammation, and oxidative stress. These metabolic derangements can promote lipotoxicity, impair vascular function, and ultimately increase the risk of cardiovascular events and mortality [22]. (2) Atherogenic Dyslipidemia: Elevated TyG levels have been linked to atherogenic dyslipidemia, characterized by increased levels of triglycerides and LDL-C, along with decreased HDL-C levels. This lipid profile is known to contribute to the development of atherosclerosis and subsequent cardiovascular events [23]. (3) Chronic Inflammation and Endothelial Dysfunction: TyG has been associated with chronic low-grade inflammation, as indicated by increased levels of inflammatory markers such as C-reactive protein (CRP) and interleukin-6 (IL-6) [24]. (4) Hypercoagulability and Thrombosis: Elevated TyG levels have been associated with hypercoagulability and an increased risk of thrombotic events. Dysregulated coagulation pathways and platelet activation can contribute to the formation of arterial and venous thrombosis, leading to cardiovascular events [25]. (5) Impaired beta-cell function: Low TyG index values may indicate impaired beta-cell function, which could increase the risk of hypoglycemia and adverse cardiovascular events [26].

In terms of sex differences, there are several potential biological mechanisms that could explain this phenomenon, including: (1) Hormonal Differences: Hormonal differences between males and females could contribute to the observed sex differences in the association between TyG and mortality risk. For example, estrogen has been shown to have a protective effect on the cardiovascular system, reducing the risk of atherosclerosis, thrombosis, and CVD. Conversely, androgens such as testosterone have been associated with an increased CVD risk. Therefore, the hormonal differences between males and females could explain why males are more vulnerable to the adverse effects of TyG on survival [27]. (2) Androgens and IR: Androgens are known to induce IR, which can lead to increased TyG levels, and subsequently, dyslipidemia and systemic inflammation. Studies have shown that androgens may play a critical role in the development of metabolic disorders and the subsequent increased CVD risk in men [28]. (3) Visceral Adiposity: Differences in visceral adiposity may also contribute to sex-specific differences in the U-shaped association of TyG with mortality. Men typically have a higher proportion of visceral adipose tissue, which is more metabolically active and produces more inflammatory cytokines than subcutaneous adipose tissue [29]. This may contribute to increased IR, dyslipidemia, and cardiovascular risk in men compared to women. (4) Endothelial Dysfunction and Chronic Inflammation: Men have been shown to have higher levels of chronic inflammation and endothelial dysfunction, which are critical contributors to the development of atherosclerosis and CVD. The stronger association between TyG and cardiovascular mortality observed in men could be attributed to the higher levels of chronic inflammation and endothelial dysfunction [30]. (5) Lifestyle Differences: There may also be lifestyle differences between males and females that contribute to the observed sex differences in the association between TyG and mortality risk. For example, men are more likely to engage in risky health behaviors such as smoking, excessive alcohol consumption, and a sedentary lifestyle, which can increase the CVD risk. Conversely, women tend to have healthier lifestyles, which could explain why they are less susceptible to the adverse effects of TyG on survival [31].

Some limitations in our study should be noted. First, given that this is an observational study, causal associations cannot be inferred from the results. In addition, measurement error or bias in our study cannot be ruled out. Second, the calculation of the TyG index was based on a single measurement at baseline, which may not reflect dynamic changes during long-term follow-up. Third, since our results are from the general US population, caution should be exercised in generalizing our conclusions to other ethnic groups or other populations. Fourth, although we adjusted for multiple covariates, residual or unknown confounders could not be completely ruled out.

## Conclusions

This study identified a U-shaped relationship between the TyG index and all-cause and cardiovascular mortality in the general population, with evidence of sex-based modification of this nonlinear association. Below a certain threshold, the association of the TyG index with mortality risk remained relatively consistent across genders. However, above this threshold, males exhibited a stronger adverse effect of the TyG index on survival than females. These findings suggest that clinicians should consider threshold effects and sex differences when managing TyG index levels to improve survival.

## Data Availability

The datasets generated and analyzed in the current study are available at NHANES website: https://www.cdc.gov/nchs/nhanes/index.htm.
